# Supplementary material for: Possible nitrogen fertilization of the early Earth Ocean by microbial continental ecosystems
Source: Nat Commun. 2018 Jun 28;9:2530. doi: 10.1038/s41467-018-04995-y (PMC6023897; doi:10.1038/s41467-018-04995-y)
Supplement: Supplementary file 1 — Supplementary Information [file 41467_2018_4995_MOESM1_ESM.pdf]

Thomazo et al. **Possible nitrogen fertilization of the early Earth Ocean by microbial continental ecosystems**

Supplementary Table 1: Matrix of calculated N transport fluxes to underlying soil (Tmol yr^{-1}) vs. percentage of colonized continental area for net N outputs fluxes varying from 0.0133 g N m^{-2} yr^{-1} to 8.820 g N m^{-2} yr^{-1}. The red values represent calculated result scaled to the modern export flux threshold.

N transport underlying soil (Tmol yr^{-1}) Vs.% modern continental area

Net N outputs from crusts to subsurface soil (m^{-2} yr^{-1})

Surface continent (km^2)	%	0.013	0.020	0.025	0.050	0.080	0.110	0.133	0.140	0.144	0.190	0.200	0.247	0.260	0.290
148647000	100	0.141	0.212	0.265	0.531	0.849	1.167	1.412	1.486	1.528	2.016	2.123	2.621	2.759	3.078
133782300	90	0.127	0.191	0.239	0.478	0.764	1.051	1.270	1.337	1.375	1.815	1.910	2.359	2.483	2.770
118917600	80	0.113	0.170	0.212	0.425	0.679	0.934	1.129	1.189	1.223	1.613	1.698	2.097	2.208	2.462
104052900	70	0.099	0.149	0.186	0.371	0.594	0.817	0.988	1.040	1.070	1.412	1.486	1.835	1.932	2.154
89188200	60	0.085	0.127	0.159	0.318	0.509	0.700	0.847	0.891	0.917	1.210	1.274	1.573	1.656	1.847
74323500	50	0.071	0.106	0.133	0.265	0.425	0.584	0.706	0.743	0.764	1.008	1.061	1.311	1.380	1.539
59458800	40	0.056	0.085	0.106	0.212	0.340	0.467	0.565	0.594	0.611	0.807	0.849	1.049	1.104	1.231
44594100	30	0.042	0.064	0.080	0.159	0.255	0.350	0.423	0.446	0.458	0.605	0.637	0.786	0.828	0.923
29729400	20	0.028	0.042	0.053	0.106	0.170	0.233	0.282	0.297	0.306	0.403	0.425	0.524	0.552	0.616
25269990	17	0.024	0.036	0.045	0.090	0.144	0.198	0.240	0.253	0.260	0.343	0.361	0.446	0.469	0.523
14864700	10	0.014	0.021	0.027	0.053	0.085	0.117	0.141	0.149	0.153	0.202	0.212	0.262	0.276	0.308
13378230	9	0.013	0.019	0.024	0.048	0.076	0.105	0.127	0.134	0.138	0.181	0.191	0.236	0.248	0.277
11891760	8	0.011	0.017	0.021	0.042	0.068	0.093	0.113	0.119	0.122	0.161	0.170	0.210	0.221	0.246
10405290	7	0.010	0.015	0.019	0.037	0.059	0.082	0.099	0.104	0.107	0.141	0.149	0.184	0.193	0.215
8918820	6	0.008	0.013	0.016	0.032	0.051	0.070	0.085	0.089	0.092	0.121	0.127	0.157	0.166	0.185
7432350	5	0.007	0.011	0.013	0.027	0.042	0.058	0.071	0.074	0.076	0.101	0.106	0.131	0.138	0.154
5945880	4	0.006	0.008	0.011	0.021	0.034	0.047	0.056	0.059	0.061	0.081	0.085	0.105	0.110	0.123
4459410	3	0.004	0.006	0.008	0.016	0.025	0.035	0.042	0.045	0.046	0.060	0.064	0.079	0.083	0.092
2972940	2	0.003	0.004	0.005	0.011	0.017	0.023	0.028	0.030	0.031	0.040	0.042	0.052	0.055	0.062
1486470	1	0.001	0.002	0.003	0.005	0.008	0.012	0.014	0.015	0.015	0.020	0.021	0.026	0.028	0.031
0	0	0.000	0.000	0.000	0.000	0.000	0.000	0.000	0.000	0.000	0.000	0.000	0.000	0.000	0.000

Surface continent (km^2)	%	0.320	0.342	0.380	0.410	0.440	0.470	0.500	0.530	0.560	0.590	0.620	0.650	0.680	0.710	0.740	0.770	0.800	1.900	2.080	8.820
148647000	100	3.396	3.630	4.033	4.351	4.670	4.988	5.307	5.625	5.943	6.262	6.580	6.899	7.217	7.535	7.854	8.172	8.490	20.165	22.075	93.607
133782300	90	3.057	3.267	3.630	3.916	4.203	4.489	4.776	5.062	5.349	5.636	5.922	6.209	6.495	6.782	7.068	7.355	7.641	18.148	19.868	84.247
118917600	80	2.717	2.904	3.226	3.481	3.736	3.991	4.245	4.500	4.755	5.009	5.264	5.519	5.774	6.028	6.283	6.538	6.792	16.132	17.660	74.886
104052900	70	2.377	2.541	2.823	3.046	3.269	3.492	3.715	3.937	4.160	4.383	4.606	4.829	5.052	5.275	5.498	5.720	5.943	14.115	15.453	65.525
89188200	60	2.038	2.178	2.420	2.611	2.802	2.993	3.184	3.375	3.566	3.757	3.948	4.139	4.330	4.521	4.712	4.903	5.094	12.099	13.245	56.164
74323500	50	1.698	1.815	2.016	2.176	2.335	2.494	2.653	2.812	2.972	3.131	3.290	3.449	3.608	3.768	3.927	4.086	4.245	10.082	11.038	46.804
59458800	40	1.358	1.452	1.613	1.741	1.868	1.995	2.123	2.250	2.377	2.505	2.632	2.759	2.887	3.014	3.141	3.269	3.396	8.066	8.830	37.443
44594100	30	1.019	1.089	1.210	1.305	1.401	1.496	1.592	1.687	1.783	1.879	1.974	2.070	2.165	2.261	2.356	2.452	2.547	6.049	6.623	28.082
29729400	20	0.679	0.726	0.807	0.870	0.934	0.998	1.061	1.125	1.189	1.252	1.316	1.380	1.443	1.507	1.571	1.634	1.698	4.033	4.415	18.721
25269990	17	0.577	0.617	0.686	0.740	0.794	0.848	0.902	0.956	1.010	1.064	1.119	1.173	1.227	1.281	1.335	1.389	1.443	3.428	3.753	15.913
14864700	10	0.340	0.363	0.403	0.435	0.467	0.499	0.531	0.562	0.594	0.626	0.658	0.690	0.722	0.754	0.785	0.817	0.849	2.016	2.208	9.361
13378230	9	0.306	0.327	0.363	0.392	0.420	0.449	0.478	0.506	0.535	0.564	0.592	0.621	0.650	0.678	0.707	0.735	0.764	1.815	1.987	8.425
11891760	8	0.272	0.290	0.323	0.348	0.374	0.399	0.425	0.450	0.475	0.501	0.526	0.552	0.577	0.603	0.628	0.654	0.679	1.613	1.766	7.489
10405290	7	0.238	0.254	0.282	0.305	0.327	0.349	0.371	0.394	0.416	0.438	0.461	0.483	0.505	0.527	0.550	0.572	0.594	1.412	1.545	6.553
8918820	6	0.204	0.218	0.242	0.261	0.280	0.299	0.318	0.337	0.357	0.376	0.395	0.414	0.433	0.452	0.471	0.490	0.509	1.210	1.325	5.616
7432350	5	0.170	0.181	0.202	0.218	0.233	0.249	0.265	0.281	0.297	0.313	0.329	0.345	0.361	0.377	0.393	0.409	0.425	1.008	1.104	4.680
5945880	4	0.136	0.145	0.161	0.174	0.187	0.200	0.212	0.225	0.238	0.250	0.263	0.276	0.289	0.301	0.314	0.327	0.340	0.807	0.883	3.744
4459410	3	0.102	0.109	0.121	0.131	0.140	0.150	0.159	0.169	0.178	0.188	0.197	0.207	0.217	0.226	0.236	0.245	0.255	0.605	0.662	2.808
2972940	2	0.068	0.073	0.081	0.087	0.093	0.100	0.106	0.112	0.119	0.125	0.132	0.138	0.144	0.151	0.157	0.163	0.170	0.403	0.442	1.872
1486470	1	0.034	0.036	0.040	0.044	0.047	0.050	0.053	0.056	0.059	0.063	0.066	0.069	0.072	0.075	0.079	0.082	0.085	0.202	0.221	0.936
0	0	0.000	0.000	0.000	0.000	0.000	0.000	0.000	0.000	0.000	0.000	0.000	0.000	0.000	0.000	0.000	0.000	0.000	0.000	0.000	0.000

range of direct estimates

Johnson et al.[1]

Thiet et al.[2]

Rychert and Skujins[3]; West and Skujins[4]

Evans and Johansen[5]

range of indirect estimates

Rychert et al.[6]

Evans and Lange[7]; Belnap[8,9]; Russow et al.[10]; Stewart et al.[11-13]; Caputa et al.[14]

Elbert et al.[15]

N transport underlying soil (Tmol yr^{-1}) Vs % Archean continental area

Net N outputs from crusts to subsurface soil (m^{-2} yr^{-1})

Surface continent (km^2)	%	0.013	0.020	0.025	0.050	0.080	0.110	0.133	0.140	0.144	0.190	0.200	0.247	0.260	0.290
89188200	100	0.085	0.127	0.159	0.318	0.509	0.700	0.847	0.891	0.917	1.210	1.274	1.573	1.656	1.847
80269380	90	0.076	0.115	0.143	0.287	0.458	0.630	0.762	0.802	0.825	1.089	1.146	1.416	1.490	1.662
71350560	80	0.068	0.102	0.127	0.255	0.408	0.560	0.678	0.713	0.734	0.968	1.019	1.258	1.325	1.477
62431740	70	0.059	0.089	0.111	0.223	0.357	0.490	0.593	0.624	0.642	0.847	0.891	1.101	1.159	1.293
53512920	60	0.051	0.076	0.096	0.191	0.306	0.420	0.508	0.535	0.550	0.726	0.764	0.944	0.993	1.108
44594100	50	0.042	0.064	0.080	0.159	0.255	0.350	0.423	0.446	0.458	0.605	0.637	0.786	0.828	0.923
35675280	40	0.034	0.051	0.064	0.127	0.204	0.280	0.339	0.357	0.367	0.484	0.509	0.629	0.662	0.739
26756460	30	0.025	0.038	0.048	0.096	0.153	0.210	0.254	0.267	0.275	0.363	0.382	0.472	0.497	0.554
17837640	20	0.017	0.025	0.032	0.064	0.102	0.140	0.169	0.178	0.183	0.242	0.255	0.315	0.331	0.369
15161994	17	0.014	0.022	0.027	0.054	0.087	0.119	0.144	0.152	0.156	0.206	0.217	0.267	0.281	0.314
8918820	10	0.008	0.013	0.016	0.032	0.051	0.070	0.085	0.089	0.092	0.121	0.127	0.157	0.166	0.185
8026938	9	0.008	0.011	0.014	0.029	0.046	0.063	0.076	0.080	0.083	0.109	0.115	0.142	0.149	0.166
7135056	8	0.007	0.010	0.013	0.025	0.041	0.056	0.068	0.071	0.073	0.097	0.102	0.126	0.132	0.148
6243174	7	0.006	0.009	0.011	0.022	0.036	0.049	0.059	0.062	0.064	0.085	0.089	0.110	0.116	0.129
5351292	6	0.005	0.008	0.010	0.019	0.031	0.042	0.051	0.053	0.055	0.073	0.076	0.094	0.099	0.111
4459410	5	0.004	0.006	0.008	0.016	0.025	0.035	0.042	0.045	0.046	0.060	0.064	0.079	0.083	0.092
3567528	4	0.003	0.005	0.006	0.013	0.020	0.028	0.034	0.036	0.037	0.048	0.051	0.063	0.066	0.074
2675646	3	0.003	0.004	0.005	0.010	0.015	0.021	0.025	0.027	0.028	0.036	0.038	0.047	0.050	0.055
1783764	2	0.002	0.003	0.003	0.006	0.010	0.014	0.017	0.018	0.018	0.024	0.025	0.031	0.033	0.037
891882	1	0.001	0.001	0.002	0.003	0.005	0.007	0.008	0.009	0.009	0.012	0.013	0.016	0.017	0.018
0	0	0.000	0.000	0.000	0.000	0.000	0.000	0.000	0.000	0.000	0.000	0.000	0.000	0.000	0.000

Surface continent (km^2)	%	0.320	0.342	0.380	0.410	0.440	0.470	0.500	0.530	0.560	0.590	0.620	0.650	0.680	0.710	0.740	0.770	0.800	2.500	2.080	8.820
89188200	100	2.038	2.178	2.420	2.611	2.802	2.993	3.184	3.375	3.566	3.757	3.948	4.139	4.330	4.521	4.712	4.903	5.094	12.099	13.245	56.164
80269380	90	1.834	1.960	2.178	2.350	2.522	2.694	2.866	3.037	3.209	3.381	3.553	3.725	3.897	4.069	4.241	4.413	4.585	10.889	11.921	50.548
71350560	80	1.630	1.742	1.936	2.089	2.241	2.394	2.547	2.700	2.853	3.006	3.158	3.311	3.464	3.617	3.770	3.923	4.075	9.679	10.596	44.932
62431740	70	1.426	1.524	1.694	1.828	1.961	2.095	2.229	2.362	2.496	2.630	2.764	2.897	3.031	3.165	3.299	3.432	3.566	8.469	9.272	39.315
53512920	60	1.223	1.307	1.452	1.566	1.681	1.796	1.910	2.025	2.140	2.254	2.369	2.483	2.598	2.713	2.827	2.942	3.057	7.259	7.947	33.699
44594100	50	1.019	1.089	1.210	1.305	1.401	1.496	1.592	1.687	1.783	1.879	1.974	2.070	2.165	2.261	2.356	2.452	2.547	6.049	6.623	28.082
35675280	40	0.815	0.871	0.968	1.044	1.121	1.197	1.274	1.350	1.426	1.503	1.579	1.656	1.732	1.808	1.885	1.961	2.038	4.840	5.298	22.466
26756460	30	0.611	0.653	0.726	0.783	0.841	0.898	0.955	1.012	1.070	1.127	1.184	1.242	1.299	1.356	1.414	1.471	1.528	3.630	3.974	16.849
17837640	20	0.408	0.436	0.484	0.522	0.560	0.599	0.637	0.675	0.713	0.751	0.790	0.828	0.866	0.904	0.942	0.981	1.019	2.420	2.649	11.233
15161994	17	0.346	0.370	0.411	0.444	0.476	0.509	0.541	0.574	0.606	0.639	0.671	0.704	0.736	0.769	0.801	0.834	0.866	2.057	2.252	9.548
8918820	10	0.204	0.218	0.242	0.261	0.280	0.299	0.318	0.337	0.357	0.376	0.395	0.414	0.433	0.452	0.471	0.490	0.509	1.210	1.325	5.616
8026938	9	0.183	0.196	0.218	0.235	0.252	0.269	0.287	0.304	0.321	0.338	0.355	0.373	0.390	0.407	0.424	0.441	0.458	1.089	1.192	5.055
7135056	8	0.163	0.174	0.194	0.209	0.224	0.239	0.255	0.270	0.285	0.301	0.316	0.331	0.346	0.362	0.377	0.392	0.408	0.968	1.060	4.493
6243174	7	0.143	0.152	0.169	0.183	0.196	0.210	0.223	0.236	0.250	0.263	0.276	0.290	0.303	0.316	0.330	0.343	0.357	0.847	0.927	3.932
5351292	6	0.122	0.131	0.145	0.157	0.168	0.180	0.191	0.202	0.214	0.225	0.237	0.248	0.260	0.271	0.283	0.294	0.306	0.726	0.795	3.370
4459410	5	0.102	0.109	0.121	0.131	0.140	0.150	0.159	0.169	0.178	0.188	0.197	0.207	0.217	0.226	0.236	0.245	0.255	0.605	0.662	2.808
3567528	4	0.082	0.087	0.097	0.104	0.112	0.120	0.127	0.135	0.143	0.150	0.158	0.166	0.173	0.181	0.188	0.196	0.204	0.484	0.530	2.247
2675646	3	0.061	0.065	0.073	0.078	0.084	0.090	0.096	0.101	0.107	0.113	0.118	0.124	0.130	0.136	0.141	0.147	0.153	0.363	0.397	1.685
1783764	2	0.041	0.044	0.048	0.052	0.056	0.060	0.064	0.067	0.071	0.075	0.079	0.083	0.087	0.090	0.094	0.098	0.102	0.242	0.265	1.123
891882	1	0.020	0.022	0.024	0.026	0.028	0.030	0.032	0.034	0.036	0.038	0.039	0.041	0.043	0.045	0.047	0.049	0.051	0.121	0.132	0.562
0	0	0.000	0.000	0.000	0.000	0.000	0.000	0.000	0.000	0.000	0.000	0.000	0.000	0.000	0.000	0.000	0.000	0.000	0.000	0.000	0.000

Supplementary references:

1. Johnson, S. L., Neuer, S., Garcia-Pichel., F. Export of nitrogenous compounds due to incomplete cycling within biological soil crusts of arid lands *Environ. Microbiol.* **9**, 680 (2007).

2. Thiet, R. K., Boerner, R. E., Nagy, M. & Jardine, R. The effect of biological soil crusts on throughput of rainwater and N into Lake Michigan sand dune soils. *Plant and soil* ***278***, 235-251 (2005).

3. Rychert, R. C. & Skujins, J. Nitrogen fixation by blue-green algae-lichen crusts in the Great Basin Desert. *Soil Sci. Soc. Am. Proc*. **38**, 768–771 (1974).

4. West, N.E. & Skujins, J. The nitrogen cycle in North American cold-winter semi-desert ecosystems. *Oecologia* **12**, 45–53 (1977).

5. Evans, R.D. & Johansen, J.R. Microbiotic crusts and ecosystem processes. *Crit. Rev. Plant Sci.* **18**, 183–225 (1999).

6. Rychert, R.C., Skukins, J., Sorensen, D. & Porcella, D. Nitrogen fixation by lichens and free-living microorganisms in deserts. In: *Nitrogen in Desert Ecosystems Dowden*. Ed. N E J J West Skujins pp. 20–30. Hutchinson, and Ross, Inc, Stroudsburg (1978).

7. Evans, R.D. & Lange, O.L. Biological soil crusts and ecosystem nitrogen and carbon dynamics. In: *Biological Soil Crusts: Structure, Function, and Management, Ecological Studies*, Vol. 150. Eds. J Belnap and O L Lange pp. 264–279. Springer-Verlag, Berlin. (2001)

8. Belnap, J. Nitrogen fixation in biological soil crusts from southeast Utah, USA. *Biol. Fertil. Soils* **35**, 128–135 (2002).

9. Belnap, J. Impacts of off road vehicles on nitrogen cycles in biological soil crusts: resistance in different US deserts. *J. Arid Environ.* **52**, 155–165 (2002).

10. Russow, R., Veste, M. & B**ö**hme, F. A natural ^{15}N approach to determine the biological fixation of atmospheric nitrogen by biological soil crusts of the Negev Desert. *Rapid Commun. Mass Spectrom.* **19**, 3451–3456 (2005).

11. Stewart, K.J., Coxson, D. & Grogan, P. Nitrogen inputs by associative cyanobacteria across a low arctic tundra landscape. *Arct. Antarct. Alp. Res*. **43**, 267–278. (2011)

12. Stewart, K.J., Coxson, D. & Siciliano, S.D. Small-scale spatial patterns in N_2-fixation and nutrient availability in an arctic hummock–hollow ecosystem. *Soil Biol. Biochem.* **43**, 133–140 (2011).

13. Stewart, K.J., Lamb, E.G., Coxson, D.S. & Siciliano, S.D. Bryophyte-cyanobacterial associations as a key factor in N_2-fixation across the Canadian Arctic. *Plant Soil* **344**, 335–346 (2011)

14. Caputa, K., Coxson, D. & Sanborn, P. Seasonal patterns of nitrogen fixation in biological soil crusts from British Columbia's Chilcotin grasslands. *Botany* **641**, 631–641 (2013).

15. Elbert, W. et al. Contribution of cryptogamic covers to the global cycles of carbon and nitrogen. *Nature Geosci.* **5**, 459–462 (2012).
